# Adipose-Derived Stem Cells Promote Bone Coupling in Bisphosphonate-Related Osteonecrosis of the Jaw by TGF-β1

**DOI:** 10.3389/fcell.2021.639590

**Published:** 2021-05-12

**Authors:** Xian Dong, Linhai He, Xiaolong Zang, Yang He, Jingang An, Baoping Wu, Xinhua Liu, Hongsen Bi, Yi Zhang, E. Xiao

**Affiliations:** ^1^Department of Oral and Maxillofacial Surgery, Peking University School and Hospital of Stomatology, Beijing, China; ^2^First Clinical Division, Peking University School and Hospital of Stomatology, Beijing, China; ^3^The First People’s Hospital of Jinzhong, Jinzhong, China; ^4^Department of Plastic Surgery, Peking University Third Hospital, Beijing, China

**Keywords:** adipose-derived mesenchymal stem cells, transforming growth factor beta1, zoledronic acid, bone remodeling, osteoclasts, bisphosphonate-related osteonecrosis of the jaw (BRONJ)

## Abstract

This study aimed to investigate molecularly targeted therapy to revive bone remodeling and prevent BRONJ by local adipose-derived stem cells (ADSCs) transplantation. Clinical samples of BRONJ and healthy jawbones were used to examine the bone coupling-related cells and TGF-β1 expression. Bone coupling-related cells and TGF-β1 expression were also assessed in BRONJ-like animal model to confirm the results in clinical samples. ADSCs were locally administered *in vivo* and the therapeutic effects were evaluated by gross observation, radiological imaging, and histological examination. Furthermore, ADSCs-conditioned medium (ADSCs-CM) and neutralizing antibody were applied to assess the effects of ADSCs-derived TGF-β1 on restoring bone coupling *in vivo*. Osteoclast formation and resorption assays were performed to evaluate the effects of ADSCs-derived TGF-β1 on ZA-treated pre-osteoclasts. Cell migration was performed to assess the effects of ADSCs-derived TGF-β1 on patients’ bone marrow stem cells (BMSCs). The number of osteoclasts, Runx2-positive bone-lining cells (BLCs) and TGF-β1 expression were decreased in BRONJ and animal model jaw bone samples. These reductions were significantly rescued and necrotic jawbone healing was effectively promoted by local ADSCs administration in BRONJ-like animal models. Mechanistically, ADSCs-CM mainly contributed to promoting bone coupling, while TGF-β1 neutralizing antibody in the conditioned medium inhibited these effects. Besides, osteoclastogenesis and patients’ BMSCs migration were also rescued by ADSCs-derived TGF-β1. Furthermore, bone resorption-released bone matrix TGF-β1, together with ADSCs-derived TGF-β1, synergistically contributed to rescuing BMSCs migration. Collectively, ADSCs promoted bone healing of BRONJ by TGF-β1-activated osteoclastogenesis and BMSCs migration capacities.

## Introduction

Bisphosphonate-related osteonecrosis of the jaw (BRONJ) has become a predominant side-effect of bisphosphonate therapy which is often administered to patients with malignant disease (Ruggiero et al., 2014). It has been estimated that from 2004–2014, BRONJ has affected over 11,440–17,160 patients/year worldwide, and the incidence is still rising (Khan et al., 2015; Silva et al., 2016). Its severe clinical symptoms may cause marked limitations in the quality of life, such as persistent extraction sockets exposure, aching bone pain in the jaw, or even numbness of the associated branch of the trigeminal nerve (Khan et al., 2015). The diagnostic criteria provided by the American Association of Oral and Maxillofacial Surgeons were up to date in 2014. Exposed jaw bone and long medical history were clearly defined. To date, BRONJ is still a big challenge for oral and maxillafacial surgeon due to the not quite clear pathological mechanisms. Though deficiency in osteoclasts and bone remodeling is considered one of the most important mechanisms (Reid, 2009; Ruggiero et al., 2014), there are still no much effective therapies to prevent BRONJ.

Recent studies had suggested that allogeneic mesenchymal stem cells (MSCs) i.v. was effective for treating BRONJ-like disease models (Kikuiri et al., 2010; Li et al., 2013). However, many patients with intractable BRONJ are suffering cancer and systemic MSCs application may increase risks of disseminated intravascular thrombosis, and cancer metastasis or recurrence caused by MSCs-induced immunosuppression (Valeta-Magara et al., 2019). Our previous serial study reported the impaired function of MSCs in bone lesions and surrounding tissues (He et al., 2017b) and local administration of adipose MSCs promoted bone regeneration (Zang et al., 2019). However, whether local administration of adipose MSCs could rescue MSCs capacities from bone lesions and bone coupling process is not clear.

TGF-β1, a member of the transforming growth factor beta superfamily, has multifunctional regulatory properties in bone component cells: it enhances osteoblast proliferation and osteoclast maturation and mediates bone homeostasis (Kasagi and Chen, 2013; Wu et al., 2016). Therefore, TGF-β1 can act as a mediator that couples osteogenic and osteoclastogenic cells for bone metabolism. However, whether TGF-β1 plays a role in reviving of bone remodeling of BRONJ bone lesion during ADSCs local application is still unknown.

In this study, expression of TGF-β1 was evaluated in bone samples from BRONJ patients and the BRONJ-like animal model. Furthermore, the effects of adipose-derived stem cells (ADSCs) on rescuing bone coupling in the BRONJ-like model were investigated. The ADSCs-conditioned medium (ADSCs-CM) and TGF-β1 neutralizing antibody were used to explore the molecular mechanisms. *In vitro*, osteoclast formation, resorption, and BMSCs migration assays were used to evaluate the effect of ADSC-secreted TGF-β1 on osteoclasts capacities, and BMSCs migration ability.

## Materials and Methods

### Animals

In this study, 44 rabbits were used. 32 of which were randomly divided into four groups to assess the ADSCs effects on rescuing bone coupling of BRONJ. These groups included BRONJ lesion group (*n* = 8): induced BRONJ like jaw bone necrosis without any treatment, abbreviated as ZA(+)HA(−)ADSC(−). Hydroxyapatite-treated group (*n* = 8): induced BRONJ like jaw bone necrosis treated with hydroxyapatite (HA, Beijing YHJ Science and Trade Co. Ltd.), abbreviated as ZA(+)HA(+)ADSC(−), ADSCs-treated group (*n* = 8) treated with ADSCs mixed with hydroxyapatite to evaluate the effects of ASDCs on preventing BRONJ, abbreviated as ZA(+)HA(+)ADSC(+), and the blank control group (*n* = 8) only saline administration as the physiological tooth socket healing group, abbreviated as ZA(−)HA(−)ADSC(−). Half of the animals in each group were sacrificed by anesthesia overdose at 2- and 8-weeks post-teeth extraction, and samples were collected for subsequent analysis. The remaining 12 of 44 rabbits were randomly divided into 2 groups to explore the role of ADSCs-derived TGF-β1 in rescuing bone coupling of BRONJ. ADSCs-CM treated group (*n* = 6), induced BRONJ like jaw bone necrosis treated with mixture of ADSCs-CM and hydroxyapatite to evaluate the effect of ADSCs-CM on rescuing bone coupling of BRONJ, abbreviated as ZA(+)HA(+)ADSCs-CM(+)TGF-β1-NAb(−) group and TGF-β1 neutralizing antibody-treated group (*n* = 6) treated with a mixture of ADSCs-CM, hydroxyapatite, and TGF-β1 neutralizing antibody to assess the role of TGF-β1 derived from ADSCs-CM in rescuing bone coupling of BRONJ, abbreviated as ZA(+)HA(+)ADSCs-CM(+)TGF-β1-NAb(+) group. These two animal groups were sacrificed by overdose anesthesia at 8 weeks post-teeth extraction, and samples were collected for subsequent analysis. All animal experiments were performed under an institutionally approved protocol for animal research by the Ethics Committee of the Peking University Health Science Center (LA2018017). Animals had unlimited access to food and water. The rabbits were anesthetized by intravenous (iv.) injection of 2% sodium pentobarbital (20mg/kg, P3761, Sigma, United States) and xylazine (50 μg/kg, Jilin Huamu Animal Health Products Co., Ltd. China) during the tooth extraction.

### Patients

Five BRONJ patients, diagnosed according to AAMOS criteria 2014, were included in this study whose detailed clinical information is listed in Supplementary Table 1. Patients’ bone samples were obtained during the surgeries at Peking University School and Hospital of Stomatology. Healthy control bone samples were obtained from five donors who had undergone orthopedic surgery. The study was approved by the Ethics Committee of the Peking University Health Science Center (IRB0000105211002), and written informed consent was obtained from all participants.

### Induction of BRONJ-Like Animal Model

The rabbit model of BRONJ was induced as we previously reported (Zang et al., 2019). Detailed methods are described in the Supplementary File 2.

### Isolation and Culture of Human BMSCs

Human mandibular bones of BRONJ patients or healthy donors were collected at the Peking University Hospital of Stomatology, approved by the Ethics Committee of Peking University (IRB00001052-11002). After surgery, bone biopsies were thoroughly cut into 1-mm^3^ cubes and digested with dispase II (4 mg/mL) and collagenase I (2 mg/mL) for 30 min at 37°C. Cells were collected by centrifugation at 1,200 rpm for 5 min, then filtered, resuspended and seeded in a 100-mm culture dish (Corning, NY, United States). Following overnight culture at 37°C in a humidified atmosphere of 5% CO_2_, unattached cells were discarded and the medium was changed every 2 days. Cells were maintained at 37°C and 5% CO_2_. BMSCs at passage 3 were used for the migration assay.

### Transplantation of Human ADSCs

Adipose-derived stem cells used in this study were from our ADSCs bank as described in our previous study (Zang et al., 2019). As previously reported, ADSCs (5 × 10^6^ cells/300 μL of FBS-free α-MEM) were mixed with 40 mg of HA (particle size: 0.5–1.0 mm, Beijing YHJ Science and Trade Co. Ltd.) and incubated at 37°C for 30 min. The mixture was locally implanted in the tooth extraction sites for each side of alveolar sockets under general anesthesia.

### Immunochemistry

Immunohistochemical staining (IHC) of the jaw samples from BRONJ patients and healthy donors (*n* = 5 per group) and alveolar socket samples from all animal groups (in ADSCs therapy experiment, *n* = 4 per group per time point; in second animal model batch ADSCs-CM therapy experiment, *n* = 6 per group) was performed with anti-Runx2 (Abcam, ab76956, United States) and anti-TGF-β1 antibodies as previously reported (Liang et al., 2016). Detailed methods are described in the Supplementary File 2.

### Collection of ADSCs-CM

Conditioned medium (CM) was obtained from human ADSCs at passage 3–5 (Gnecchi and Melo, 2009). Detailed methods are described in the Supplementary File 2.

### *In vitro* Osteoclastogenesis Assay

Bone marrow−derived monocytes from the tibia and femur of 6–8 week-old C57BL/6J mice were flushed with α-MEM (Gibco). Osteoclast precursors were prepared as described (He et al., 2017a). The osteoclast medium was prepared by supplementing α-MEM with macrophage colony-stimulating factor (M-CSF, R&D, 416 ML) (30 ng/ml) and receptor activator of nuclear factor kappa B ligand (RANKL, R&D, 462-TR) (50 ng/ml). The flushed bone marrow cells were plated in the 100 mm non-tissue culture dish for one night. The floating cells were collected and served as bone marrow monocytes. To induce bone marrow macrophages, monocytes were pre-cultured with 10 ng/ml of M-CSF with or without zoledronate (1 μM) for 2 days. Adherent cells were collected, suspended in α-MEM containing antibiotics and 10% FBS, counted, seeded in a 96-well plate at 5 × 10^5^ cells/well, and cultured in the osteoclast medium for 3 days to induce osteoclasts. A total of four groups were involved in this assay:

(1) Blank control group consisted of osteoclast precursors cultured in the osteoclast medium.
(2) Osteoclast precursors pretreated with zoledronate were cultured in osteoclast medium to evaluate the inhibitory effects of zoledronate on osteoclastogenesis.
(3) ADSCs-CM treated group was generated by osteoclast precursors pretreated with zoledronate, and cultured in osteoclast medium mixed with ADSCs-CM to evaluate its effects of ADSCs-CM on osteoclastogenesis, and
(4) TGF-β1 neutralizing antibody treated group was generated by osteoclast precursors which were pretreated with zoledronate, cultured in osteoclast medium mixed with ADSCs-CM and TGF-β1 neutralizing antibody to assess the role of ADSCs-derived TGF-β1 in osteoclastogenesis.

In the last two groups, osteoclast precursors pretreated with zoledronate were seeded in a 96-well plate at 5 × 10^5^ cells/well, and cultured in the osteoclast medium mixed at 1:1 ratio with ADSCs-CM in F12/DMEM with or without TGF-β1 neutralizing antibody for 5 days. In the blank control group and zoledronate pretreated group, osteoclast precursors or zoledronate pretreated osteoclast precursors were cultured in osteoclast medium mixed with FBS-free F12/DMEM at 1:1 ratio. For the identification of osteoclasts, TRAP staining was performed according to the manufacturer’s instructions (Sigma−Aldrich, St. Louis, MO, United States).

### *In vitro* Bone Resorption Assay and Bone Resorption Conditioned Medium Collection

Sixteen bovine bone slices were randomly divided into four groups:

(1) Blank control group (*n* = 4), in which osteoclast precursors were seeded on bovine slices and cultured in osteoclast medium for 7 days to assess the osteoclast resorption ability, and was abbreviated as ZA(−)ADSCs-CM(−)TGF-β1 NAb(−) group,
(2) Zoledronate-treated group (*n* = 4), in which osteoclast precursors were seeded on bovine slices and pretreated with zoledronate and cultured in osteoclast medium for 7 days to assess the inhibitory effects of zoledronate on osteoclast resorption ability, and was abbreviated as ZA(+)ADSCs-CM(−)TGF-β1 NAb(−) group,
(3) ADSCs-CM-treated group (*n* = 4), in which osteoclast precursors were seeded on bovine slices pretreated with zoledronate and cultured in osteoclast medium mixed with ADSCs-CM for 7 days to assess the effects of ADSCs-CM on rescuing osteoclast resorption ability, and was abbreviated as ZA(+)ADSCs-CM(+)TGF-β1 NAb(−) group, and
(4) TGF-β1 neutralizing antibody-treated group (*n* = 4), in which osteoclast precursors were seeded on bovine slices pretreated with zoledronate, and cultured in osteoclast medium mixed with ADSCs-CM and TGF-β1 neutralizing antibody for 7 days to assess the effects of ADSCs-CM-derived TGF-β1 on rescuing osteoclast resorption ability, and was abbreviated as ZA(+)ADSCs-CM(+)TGF-β1 NAb(+) group.

The groups 2–4 were pretreated with sterile saline overnight and the osteoclast precursors were seeded on the surface of bone slices at a density of 15 × 10^4^/well in 24-well plates and cultured in osteoclast medium for 7 days. Subsequently, cells were removed, and the slices were stained with hematoxylin and the resorbed areas were quantified as described previously (He et al., 2017a). The total number of cells and areas of resorption pits were quantified and compared using Image J software 6.0 (National Institutes of Health). The bone resorption conditioned medium (BRCM) from ZA(−)ADSCs-CM(−)TGF-β1 NAb(−) and ZA(+)ADSCs-CM(−)TGF-β1 NAb(−) groups were separately collected as non-zoledronate-treated BRCM and zoledronate-treated BRCM. Then the collected BRCMs were used to assess the migration ability of human BMSCs.

### TRAP Staining

TRAP staining (Sigma-Aldrich, St. Louis, MO, United States) was performed according to the manufacturer’s instructions. The osteoclasts were counted within the bone marrow from 3 random views per section and averaged by the total length of the bone marrow’s circumference via a BIOQUANT OSTEO Bone Biology Research System (BIOQUANT Image Analysis Corporation, Nashville, TN, United States). The osteoclast number per bone marrow circumference (i.e., per circumference of bone marrow in millimeters) was reported for each group.

### *In vitro* BMSCs Migration Assay

To explore the effects of ADSCs-derived TGF-β1 on BMSCs migration ability, the migration assay was divided into four groups:

(1) Positive control group (*n* = 4) in which healthy BMSCs were seeded and cultured in the BMSCs medium,
(2) Negative control group (*n* = 4) in which BRONJ BMSCs were seeded and cultured in the BMSCs medium,
(3) ADSCs-CM-treated group (*n* = 4) in which BRONJ BMSCs were seeded and cultured in ADSCs-CM mixed with the BMSCs medium to evaluate its effects on recovering BRONJ BMSCs’ migration ability, and
(4) TGF-β1 neutralizing antibody-treated group (*n* = 4) in which BRONJ BMSCs were seeded and cultured in the BMSCs medium mixed with ADSCs-CM and TGF-β1 neutralizing antibody to assess the role of ADSCs-derived TGF-β1 in promoting BRONJ BMSCs’ migration ability.

BMSCs from the jaws of four BRONJ patients and four healthy donors were seeded into the Culture-Insert 3 ibidi^®^ chamber (ibidi GmbH, Gräfelfing, Germany) following the manufacturer’s instructions; 5,000 cells/chamber were seeded and cultured for 12 h and then the BMSCs medium was replaced with BMSCs migration medium. Subsequently, the silicone insert devices were removed carefully and 500 μm-width cell-free gaps were created in which the cell migration could be monitored. Photographs (100× magnification) were taken at 0 and 24 h after insert devices were removed and the average size of the gaps was quantitated by Image J software (National Institutes of Health). In the ADSCs-CM-treated and TGF-β1 neutralizing antibody-treated groups, the BMSCs migration medium was consisted of F12/DMEM mixed with ADSCs-CM at 1:1 ratio with or without TGF-β1 neutralizing antibody; in positive and negative control groups, the BMSCs migration medium contained only the F12/DMEM medium. The second BMSCs migration assay was again divided into 4 groups to observe the synergistic effects of ADSCs-derived and bone resorption-released TGF-β1 on BMSCs migration ability:

(1) Control group (*n* = 4) in which healthy BMSCs were cultured in non-zoledronate-treated BRCM medium collected from *in vitro* osteoclast resorption assays.
(2) Zoledronate-treated BRCM group (*n* = 4) in which healthy BMSCs were cultured in zoledronate-treated BRCM medium collected from *in vitro* osteoclast resorption assays to evaluate the effects of zoledronate-treated BRCM on BMSCs migration ability,
(3) ADSCs-CM-treated group (*n* = 4) in which healthy BMSCs were cultured in zoledronate-treated BRCM medium mixed with ADSCs-CM to evaluate the synergistic effects of zoledronate-treated BRCM and ADSCs-CM on BMSCs migration ability, and
(4) TGF-β1 neutralizing antibody-treated group (*n* = 4) in which healthy BMSCs were cultured in zoledronate-treated BRCM medium mixed with ADSCs-CM and TGF-β1 neutralizing antibody to assess the role of TGF-β1 in the synergistic effects of zoledronate-treated BRCM and ADSCs-CM on BMSCs migration ability.

In the control and zoledronate-treated BRCM groups, the BMSCs migration medium was F12/DMEM medium mixed at 1:1 ratio with non-zoledronate-treated BRCM or zoledronate-treated BRCM. In ADSCs-CM treated and TGF-β1 neutralizing antibody-treated groups, the BMSCs migration medium was zoledronate-treated BRCM mixed at 1:1 ratio with ADSCs-CM with or without TGF-β1 neutralizing antibody. The experimental procedures in the second BMSCs migration batch were the same as the first BMSCs migration batch.

### ELISA Assay

For obtaining osteoclastic BRCM, the osteoclastic precursors were plated onto the bovine bone slices pre-treated with or without zoledronate the same as described above. The conditioned media from the osteoclast-mediated resorption were harvested at day 7, cleared for 5 min at 300 × g, and filtered through a 0.22 μm syringe filter, and the level of TGF-β1 in BRCM was detected using the ELISA kit (R&D, DB100B) according to the manufacturer’s instructions.

### RNA Isolation and Quantitative Real-Time Polymerase Chain Reaction (qRT-PCR)

Total RNA of bone in the extraction sockets or induced osteoclasts were isolated using TRIZOL reagent (15596, Invitrogen, CA). Complementary DNAs were prepared using the Go Script Reverse Transcription System (Promega). qRT-PCR was performed with the ABI Prism 7500. The primers are listed in Supplementary Tables 2, 3.

### Statistical Analysis

Student’s *t*-test and Analysis of Variance (ANOVA) were performed where applicable using IBM SPSS 22.0 software (SPSS Inc., Chicago, IL, United States). Error bars are represented as the mean ± standard error of the mean (SEM). *P*-values less than 0.05 were considered to be a significant difference.

## Results

### TGF-β1 Expression, Osteoclasts, and Runx2-Positive BLCs Are Decreased in BRONJ Patients and Rabbit Model

**FIGURE 1:**
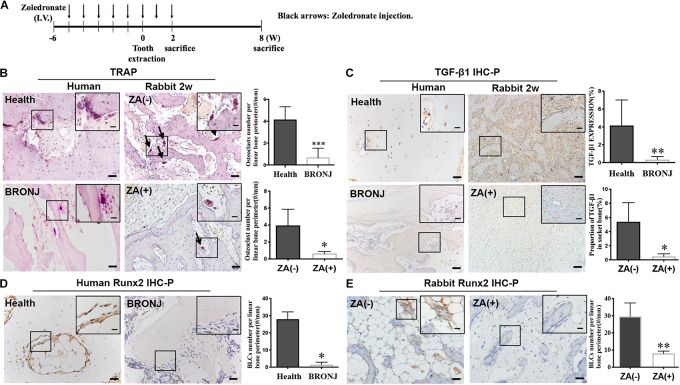
TGF-β1 and bone remodeling cells are decreased in BRONJ patients and an animal model. **(A)** Schematic diagram of inducing a BRONJ-like rabbit model. Rabbits were i.v. injected with zoledronate (800 μg/kg, black arrows) 6 weeks before tooth extraction followed by zoledronate injection for 2 weeks. **(B)** TRAP staining of osteoclast images in the jaws of BRONJ patients and healthy donors. TRAP staining of osteoclasts after 2 weeks after the surgical tooth extraction in BRONJ-like animals from zoledronic acid-untreated [ZA(–)] and zoledronic acid-treated [ZA(+)] groups. Insets show magnified boxed region. The graphs indicate the quantification of TRAP-positive cells in humans and rabbits. Black arrows show osteoclasts. **(C)** Images of TGF-β1 IHC staining and quantifying its expression in human and rabbit jaws 2 weeks after tooth extraction. **(D)** IHC of Runx2-positive BLCs and quantification in BRONJ patients and healthy donors. **(E)** IHC of Runx2 images and quantifications in rabbits in ZA(–) and ZA(+) groups. (All bars: 50 μm; bar of magnified images: 20 μm). ^∗^*p* < 0.05, ^∗∗^*p* < 0.01, ^∗∗∗^*p* < 0.005.

The rabbit model of BRONJ-like disease was induced by using zoledronate, as we previously reported (Zang et al., 2019) (Figure 1A). Two weeks after tooth extraction, TRAP staining showed that osteoclasts were decreased in the BRONJ lesion group (0.6433 ± 0.1227/mm, *n* = 4) compared to non-treated controls (3.948 ± 0.9554/mm, *n* = 4). Similarly, osteoclasts were rarely found in clinical BRONJ samples compared to healthy samples (0.3140 ± 0.2285/mm vs. 2.923 ± 0.4601/mm, *n* = 5) (Figure 1B). Furthermore, we evaluated the TGF-β1 level in the bone samples from BRONJ patients and animal models. Consistent with our previous study, TGF-β1 was suppressed in the alveolar sockets in BRONJ patients (patients vs. healthy controls, 0.3242 ± 0.1569% vs. 4.149 ± 1.278%, *n* = 5) and animal models (BRONJ lesion vs. control, 0.4796 ± 0.1935% vs. 5.388 ± 1.358%, *n* = 4) (Figure 1C). Due to TGF-β1 deficiency during bone repair, osteoclast-mediated bone remodeling might also be restricted. BLCs are known as latent osteoblasts, which cover the bone’s surface and sense alterations in bone biology. Interestingly, Runx2-positive BLCs were hardly observed on the surface of the BRONJ lesion area in the animal model (BRONJ lesion vs. control, 8.018 ± 0.7023/mm vs. 29.64 ± 3.922/mm, *n* = 4) (Figure 1E) and in the BRONJ patients’ lesions (BRONJ vs. healthy samples, 1.424 ± 0.6593/mm vs. 27.97 ± 1.909/mm, *n* = 5) (Figure 1D). These results demonstrated overall decreased skeletal cells and reduced TGF-β1 expression in BRONJ patients and animals.

### Local Implantation of ADSCs Prevents BRONJ and Rescues TGF-β1 Expression, Osteoclasts and Runx2-Positive BLCs

**FIGURE 2:**
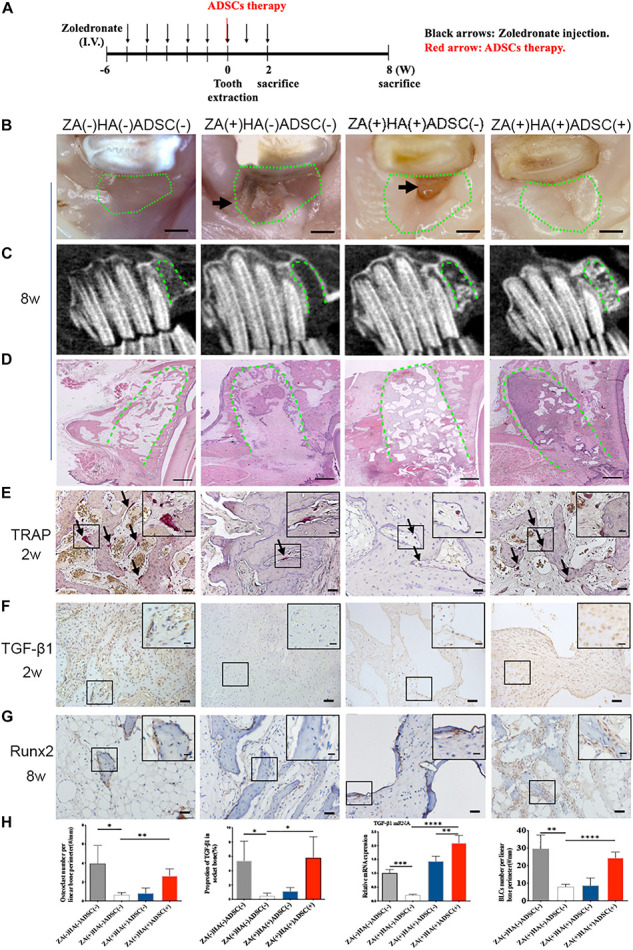
ADSCs local treatment prevents the development of BRONJ-like lesions and enhances bone remodeling in the rabbit model. **(A)** Schematic diagram of ADSCs therapy. Rabbits were i.v. injected with zoledronate (800 μg/kg, black arrows) 6 weeks before tooth extraction and ADSCs (red arrow) followed by zoledronate injection for 2 weeks. **(B)** Representative clinical appearance of gingival wounds at the extraction sites at 8 weeks after tooth extraction. Green circles represent images of the extraction sites, and black arrows indicate the exposed alveolar sockets. Bar: 1 mm. **(C)** CBCT images in each group. Green dot lines represent alveolar sockets. **(D)** H&E staining showing bone tissues in alveolar sockets (green dot line). Bar: 1 mm. **(E)** TRAP staining of alveolar socket showing osteoclasts (black arrows) in each group 2 weeks post-extraction. Insets show magnified boxed regions. Bar: 50 μm; bar of magnified image: 20 μm. **(F)** IHC showing TGF-β1 expression in alveolar sockets. Bar: 50 μm, bar of magnified image: 20 μm. **(G)** IHC of Runx2 in BLCs. Bar: 50 μm, bar of magnified image: 20 μm. **(H)** Quantification of osteoclast number, TGF-β1 expression, gene expression of TGF-β1 in bone tissue of alveolar sockets by qRT-PCR, and Runx2-positive BLCs number in each group. ZA(±), zoledronic acid-treated or untreated; HA(±), hydroxyapatite-treated or untreated; ADSCs(±), adipose-derived stem cells-treated or untreated. ^∗^*p* < 0.05, ^∗∗^*p* < 0.01, ^∗∗∗^*p* < 0.005, ^*⁣*⁣**^p < 0.0001.

Adipose-derived stem cells were locally transplanted into the zoledronate-treated animals immediately after tooth extraction to explore their therapeutic effects on BRONJ development (Figure 2A). The clinical manifestations revealed soft tissue healing in animals without zoledronate treatment and animals with HA and ADSCs treatment versus delayed gingival closure in only zoledronate-treated or HA-treated animals at 8 weeks post tooth extraction (Figure 2B). Furthermore, radiological and histological examination showed more bone regeneration at the extraction sockets after the local administration of ADSCs (Figures 2C,D). TRAP staining 2 weeks after surgical extraction showed that more osteoclasts in the ADSC-treated group (2.660 ± 0.3640/mm, *n* = 4) compared with HA (0.8064 ± 0.2748/mm, *n* = 4) or zoledronate-treated group (0.6433 ± 0.1227/mm, *n* = 4), in which osteoclast numbers were close to the group without zoledronate treatment (3.948 ± 0.9554/mm, *n* = 4) (Figure 2E). Besides increased osteoclast numbers, an increase in Runx2-positive BLCs was also observed at the newly formed bone trabecula in ADSCs-administrated animals at 8 weeks post-extraction (24.54 ± 1.596/mm vs. 8.018 ± 0.7023/mm vs. 8.709 ± 2.149/mm in ADSCs-treated group vs. Zoledronate-treated group vs. HA-treated group, *n* = 4) (Figure 2G). Moreover, TGF-β1 immunohistochemistry and qRT-PCR detected 10-fold elevated TGF-β1 expression in ADSCs-administrated tooth extraction sockets compared to other groups (Figures 2F,H), suggesting that ADSCs played a significant role in preventing BRONJ development.

### Deletion of TGF-β1 in ADSCs-CM Attenuates Osteoclastogenesis and BRONJ Healing

**FIGURE 3:**
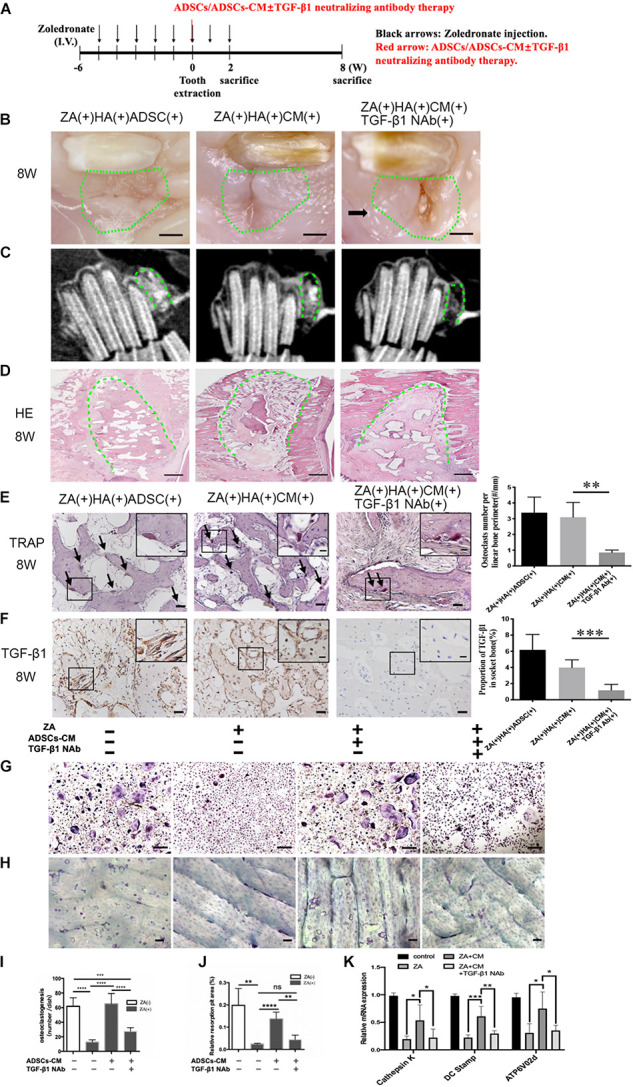
Inhibition of TGF-β1 attenuates ADSCs promoted osteoclastogenesis and BRONJ healing. **(A)** Schematic diagram of ADSCs-CM with or without TGF-β1 neutralizing antibody therapy. Rabbits were i.v. injected with zoledronate (800 μg/kg, black arrows) 6 weeks before tooth extraction and ADSCs-CM ± TGF-β1 neutralizing antibody (red arrow) treatment followed by zoledronate injection for 2 weeks. **(B)** Representative image of extraction sites 8 weeks after tooth extraction. Green circles represent images of the extraction site, and black arrows point to the exposed alveolar sockets. Bar: 1 mm. **(C)** CBCT images of each group. **(D)** Bone tissues in alveolar sockets (green dot line) were detected by H&E staining and the extent of bone tissue fraction. Bar: 1 mm. **(E)** TRAP staining in sockets at 8 weeks post-tooth extraction. Black arrows indicate osteoclasts. Insets show magnified boxed region. Bar: 50 μm, bar of magnified image: 20 μm. **(F)** IHC of TGF-β1 expression in alveolar sockets. Bar: 50 μm, bar of magnified image: 20 μm. **(G)** TRAP staining patterns of osteoclasts are shown *in vitro* and **(I)** quantitative analysis of the osteoclasts number. And **(H)** staining of bone resorption areas on bovine bone slices and **(J)** quantitative analysis of bone resorption pits. **(K)** Quantification of osteoclast differentiation-related gene expression in each group. ADSC(±), adipose-derived stem cells-treated or -untreated; CM(±), ADSCs condition medium-treated or untreated, and TGF-β1 NAb(±), ADSCs-CM with TGF-β1 neutralizing antibody-treated or -untreated. ^∗^*p* < 0.05, ^∗∗^*p* < 0.01, ^∗∗∗^*p* < 0.005, ^*⁣*⁣**^p < 0.0001.

To further investigate whether it is ADSCs secrete TGF-β1 to promote osteoclastogenesis and prevent the onset of BRONJ, we applied the ADSC-conditioned medium (ADSCs-CM) mixed with HA into the tooth extraction sockets with or without TGF-β1 neutralizing antibody (TGF-β1 NAb) (Figure 3A). Surprisingly, phenotypes similar to ADSCs local transplantation were observed in the ZA(+)HA(+)ADSCs-CM(+)TGF-β1-NAb(−) group. Furthermore, delayed mucosal wound healing was detected in ZA(+)HA(+)ADSCs-CM(+)TGF-β1-NAb(+) group compared with the ZA(+)HA(+)ADSCs-CM(+)TGF-β1-NAb(−) group after 8 weeks after tooth extraction (Figure 3B). Additionally, CBCT manifested a poorly radiopaque area after TGF-β1 NAb was added to the ADSCs-CM (Figure 3C). Histological analysis revealed that bone regeneration in the ADSCs-CM group with TGF-β1 NAb was significantly less than in the ADSCs-CM group (Figure 3D). Also, osteoclastogenesis was rescued in the ZA(+)HA(+)ADSCs-CM(+)TGF-β1-NAb(−) group (3.096 ± 0.3821/mm, *n* = 6) and abolished by adding TGF-β1 NAb to the ADSCs-CM (0.8533 ± 0.0643/mm, *n* = 6) (Figure 3E). As expected, TGF-β1 expression was up-regulated in the ZA(+)HA(+)ADSCs-CM(+)TGF-β1-NAb(−) group but suppressed after applying TGF-β1 NAb (Figure 3F).

We examined the function of TGF-β1 in ADSCs-CM-mediated osteoclastogenesis by culturing osteoclast precursors pretreated with zoledronate in ADSCs-CM with or without TGF-β1 NAb. To examine the osteoclastic function of bone resorption, osteoclast precursors were seeded on the bovine bone slices pre-treated with or without zoledronate, and then cultured in ADSCs-CM with or without TGF-β1 NAb. ADSCs-CM markedly promoted osteoclast differentiation and bone resorption capacities (0.138 ± 0.015%, *n* = 4) in the ZA(+)ADSCs-CM(+)TGF-β1 NAb(−) group, whereas the improvements were decreased when TGF-β1 NAb was applied to the ADSCs-CM (Figures 3G–J). Consistent with the above results, the osteoclast-specific genes of dendritic cell-specific transmembrane protein (DC-STAMP), cathepsin K, and ATPase H+-transporting V0 subunit d2 (Atp6v0d2) were upregulated in the ZA(+)ADSCs-CM(+)TGF-β1 NAb(−) group, while suppressed again in TGF-β1 NAb-administered group (Figure 3K). These compelling results supported the notion that ADSCs-secreted TGF-β1 has an important role in osteoclastogenesis and osteoclastic bone resorption *in vivo* and *in vitro*.

### ADSCs-Derived and Bone Resorption-Released TGF-β1 Synergetically Promotes Bone Marrow Mesenchymal Stem Cell Migration

**FIGURE 4:**
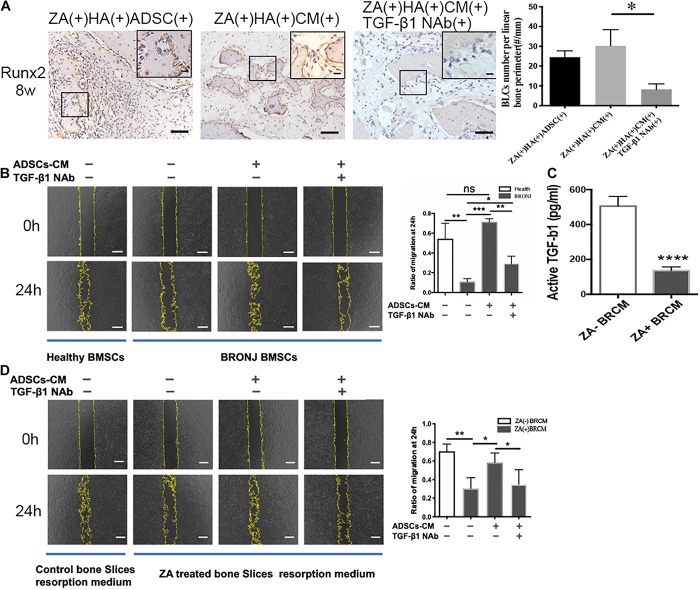
Inhibition of TGF-β1 attenuates Runx2-positive BLCs and BMSCs migration. **(A)** BLCs were evaluated by IHC of Runx-2 and quantification in alveolar sockets 8 weeks after tooth extraction in each group. The inset shows the boxed region magnified. Bar: 50 μm, bar of magnified image: 20 μm. **(B)** Migration of BMSCs from healthy individuals or BRONJ patients treated with ADSCs-CM without/with TGF-β1 NAb and its quantification. Bar: 400 μm. **(C)** TGF-β1 concentration in bone resorption supernatant was evaluated by ELISA. **(D)** Cell migration of BMSCs from healthy donors using bone resorption medium collected from different bone slices (with or without ZA-treatment) and treated with ADSCs-CM with or without TGF-β1 NAb and quantitative analysis. Osteoclastic resorption medium was collected from saline-treated bone slices control group. Bar: 400 μm. ^∗^*p* < 0.05, ^∗∗^*p* < 0.01, ^∗∗∗^*p* < 0.005, ^*⁣*⁣**^p < 0.0001.

To determine the potential role of ADSCs-secreted TGF-β1 in initiating bone coupling of BRONJ alveolar sockets, we applied ADSCs-CM and HA with or without TGF-β1 NAb into the extraction sites. As expected, ADSCs or ADSCs-CM local transplantation increased the number of Runx2-positive BLCs, which were diminished when TGF-β1 NAb was applied (30.22 ± 3.336/mm vs. 8.212 ± 1.138/mm in ADSCs-CM treated group vs. TGF-β1 NAb treated group, *n* = 6) (Figure 4A). Subsequently, we investigated whether it was ADSCs-derived TGF-β1 or bone resorption-released TGF-β1 that induced BMSCs migration. ADSCs-CM was collected to treat BMSCs from BRONJ patients and healthy donors. We found that the migratory ability of BMSCs from BRONJ patients was significantly impaired compared to those from healthy donors. Removal of TGF-β1 from the ADSCs-CM significantly inhibited the CM-induced migration (TGF-β1 NAb treated group vs. ADSCs-CM treated group, 0.2924 ± 0.0373% vs. 0.7109 ± 0.0178%, *n* = 4) (Figure 4B). These results indicated that TGF-β1 from ADSCs contributed to the increased number of Runx2-positive BLCs after ADSCs-CM or ADSCs treatment in the BRONJ-like animal model. Osteoclasts were also activated by ADSCs treatment, and TGF-β1, released from the bone matrix, was reported to play an important role during normal bone remodeling (Tang et al., 2009).

Next, we collected osteoclasts bone resorption supernatant medium (BRCM) of bovine bone slices from ZA(−)ADSCs-CM(−)TGF-β1 NAb(−) and ZA(+)ADSCs-CM(−)TGF-β1 NAb(−) groups. ELISA was employed to quantify TGF-β1 levels in these two media. The results showed that TGF-β1 was remarkably downregulated in zoledronate-treated BRCM compared to non-zoledronate treated BRCM (136.4 ± 10.51 pg/ml vs. 505.2 ± 28.09 pg/ml, *n* = 4) (Figure 4C). These two media were used to treat healthy human BMSCs and the results showed that the non-zoledronate treated BRCM induced more BMSCs to migrate to the scratched area than zoledronate-treated BRCM. Furthermore, the retarded migration of BMSCs treated with zoledronate-treated BRCM was partially rescued by supplementing with ADSCs-CM, and blocked again by applying TGF-β1 NAb to the ADSCs-CM (0.5837 ± 0.05127 vs. 0.3495 ± 0.0783, ADSCs-CM-treated group vs. TGF-β1 NAb-treated group) (Figure 4D). These results collectively indicated that local administration of ADSCs rescued the bone coupling both *in vivo* and *in vitro* in a TGF-β1-dependent manner.

## Discussion

BRONJ has been described in bisphosphonate-treated patients who suffered from persistent bone exposure for over 8 weeks and impaired bone regeneration without radiation history (Gong et al., 2017; Otto et al., 2018). The current study was conducted to elucidate the underlying stem cell therapeutic mechanism in promoting bone remodeling in BRONJ. We demonstrated that the BRONJ-like lesion in an animal model could be successfully prevented by local ADSCs transplantation, which could restore bone coupling by TGF-β1-mediated osteoclast differentiation, osteoclast resorption and BMSCs migration.

It is reported that the alveolar bone remodeling is suppressed by zoledronate treatment (Gong et al., 2017). Herein, we first analyzed the bone-coupling cells in both human samples and BRONJ animal models. Compared to healthy controls, the number of osteoclasts and Runx2-positive BLCs was markedly decreased in human BRONJ and animal model samples. However, the average age of the healthy donors was younger than the BRONJ patients. It has been shown in animal studies that the cell precursors of osteoblasts and osteoclasts reduce with increasing age (Boskey and Coleman, 2010). In clinical samples, it is challenging to get age-matched healthy controls and this age mismatch between BRONJ patients and healthy donors could potentially affect the results. To eliminate the effect of this factor, we studied samples from both patients and age-matched animal models.

BRONJ is commonly observed in patients with malignant tumors, and its treatment by stem cell transplantation is a controversial issue. Some studies have shown that systemic infusion of MSCs favors tumor growth by immunosuppression (Djouad et al., 2003) and delayed vascular clotting time (Liao et al., 2017). The treatment of BRONJ must not pose any risk that leads to inefficient anti-tumor therapy. Therefore, local transplantation of stem cells could be a promising method. Some researchers had attempted to explore the effects of local MSCs application to prevent BRONJ. Kaibuchi et al. (2016) has demonstrated bone marrow-derived multipotent mesenchymal stromal cell (MSC) local application could effectively promote wound healing in BRONJ animals. Consistently, our previous serial studies had found ADSCs local administration can effectively prevent BRONJ onset by promoting gingival wound healing (Zang et al., 2019). Moreover, our current study showed ADSCs local administration enhanced MSCs migration and rescued bone remodeling to prevent BRONJ. It is known that MSCs exert their different therapeutic effects by multi-differentiation, paracrine effects, and homing (Bateman et al., 2018). In this study, we used ADSCs and analyzed their paracrine effect by using ADSCs-CM. The results verified that ADSCs-CM had a similar therapeutic effect as ADSCs local application on bone remodeling in the BRONJ-like animal model.

The tooth socket post-extraction healing consists of three phases of inflammation, proliferation and maturation, and TGF-β1 played an essential role in tooth socket healing process (Otto et al., 2018). In the inflammatory phase, TGF-β1 functions as a chemotactic factor to attract macrophages and in the proliferative phase, it promotes the migration and differentiation of gingival fibroblasts and promote fibronectin expression (Pakyari et al., 2013; Komatsu et al., 2016). In the maturation phase, TGF-β1 plays an important role in osteoblast precursor recruitment and osteoclast maturation during bone remodeling (Kasagi and Chen, 2013). Since the wound healing of gingiva occurs prior to bone remodeling, we chose the observational timeline of bone coupling in 2 and 8 weeks post-tooth extraction as opposed to 2 weeks of gingival closure in the previous study (Zang et al., 2019).

The bone remodeling cycle contains three phases: initiation, reversal, and formation (Crane and Cao, 2014a). Bone coupling, which requires bone resorption and formation, is considered to ensue by releasing factors from the bone matrix during osteoclastic bone resorption, which then mediates the migration of BMSCs to the bone resorptive surfaces (Andersen et al., 2013; Siddiqui and Partridge, 2016). Thus, the bone coupling is a key process in bone remodeling. During the bone resorption process, growth factors, such as TGF-β1, are released from the bone matrix and couple bone resorption and formation (Tang et al., 2009; Crane and Cao, 2014b). TGF-β1 not only recruits osteoclast precursors and promotes osteoclast differentiation via RhoA (Kim et al., 2006), but also promotes BMSCs migration by Smad2 and Smad3 signaling pathway (Crane and Cao, 2014a). It has also been reported TGF-β1 dose-dependently stimulated osteoclast differentiation by receptor activator of nuclear factor kappa B ligand (RANKL) and macrophage colony-stimulating factor (M-CSF) (Kasagi and Chen, 2013). To examine if ADSCs-secreted TGF-β1 prevented the development of BRONJ, we used the ADSCs-CM with TGF-β1 neutralizing antibody both *in vivo* and *in vitro* and found that it significantly abolished the therapeutic effects of ADSCs-CM on BRONJ. We also found that osteoclastogenesis was activated mainly by ADSCs-derived TGF-β1, which upregulated DC-STAMP, cathepsin K, and Atp6v0d2. Our results as well as other studies have shown that human ADSCs were difficult to detect *in vivo* in less than 4 weeks (Park et al., 2017; Zang et al., 2019). However, the TGF-β1 level was still upregulated at 8 weeks post-extraction in the ADSCs or in the ADSCs-treated group. Therefore, it is plausible that TGF-β1 was released by activating osteoclastic bone resorption or rescuing BLCs. These results collectively indicate that ADSCs could revive osteoclast differentiation and function by secreting TGF-β1 and promote TGF-β1 release from the bone matrix to regulate bone coupling.

Quiescent BLCs constitute a significant source of osteoblasts during remodeling and can become Runx2-positive cells, which are essential for osteoblast lineage commitment and osteoblasts commitment from immature mesenchymal stromal cells (Katagiri et al., 2017; Yang et al., 2017). In our *in vivo* study, we found that the number of osteoclasts were rescued and the number of Runx2-positive BLCs increased after local implantation of ADSCs. Furthermore, we detected impaired migratory ability of BMSCs from BRONJ patients that was partially restored by ADSCs-CM and abolished again by TGF-β1 NAb *in vitro*. Furthermore, our results showed ADSCs-CM synergistically enhanced the BMSCs migration ability with osteoclast resorption conditioned medium, while TGF-β1 NAb blocked this effect. These results suggested that ADSCs-derived and osteoclast-resorption-released TGF-β1 may synergistically exert essential roles in osteoblast recruitment and subsequent bone formation during bone coupling. Therefore, a high TGF-β1 level in the ADSCs group contributes to bone regeneration in BRONJ. Taken together, ADSCs-derived TGF-β1 initially activates osteoclast differentiation, and then osteoclast-derived TGF-β1, in concert with ADSCs-secreted TGF-β1 contribute to BMSCs migration and osteogenesis. Finally, the suppressed bone coupling is rescued by the positive feedback of bone resorption and BMSCs migration.

## Conclusion

Our study suggested that ADSCs could promote extraction socket healing and bone coupling in BRONJ patients by secreting TGF-β1. These results might explain the impaired bone regeneration in BRONJ lesions and help zoledronate-treated patients with tooth extraction to prevent BRONJ.

## Data Availability Statement

The study was approved by the Ethics Committee of the Peking University Health Science Center (IRB0000105211002), and written informed consent was obtained from all participants.

## Ethics Statement

The studies involving human participants were reviewed and approved by the Ethics Committee of the Peking University Health Science Center (LA2019189). The patients/participants provided their written informed consent to participate in this study. The animal study was reviewed and approved by the Ethics Committee of the Peking University Health Science Center.

## Author Contributions

EX, YZ, LH, XD, and XZ: study design. LH, XD, XZ, YH, JA, and HB: study conduct and data collection. XD and XZ: data analysis. EX, LH, XD, and XZ: data interpretation. LH, XD, XZ, YZ, and EX: drafting manuscript. XD, XZ, LH, YZ, and EX: revising manuscript content. BW and XL: securing funding. All authors approved final version of manuscript and took responsibility for the integrity of the data analysis.

## Conflict of Interest

The authors declare that the research was conducted in the absence of any commercial or financial relationships that could be construed as a potential conflict of interest.
