# Supplementary material for: Adipose-Derived Stem Cells Promote Bone Coupling in Bisphosphonate-Related Osteonecrosis of the Jaw by TGF-β1
Source: Front Cell Dev Biol. 2021 May 12;9:639590. doi: 10.3389/fcell.2021.639590 (PMC8154543; doi:10.3389/fcell.2021.639590)
Supplement: Supplementary file 1 [file Data_Sheet_1.docx]

**Supplemental file 1**

**Table S1. Clinical features of healthy controls and patients diagnosed with bisphosphonate-induced osteonecrosis of the jaws**

| **No.** | **Age** | **Gender** | **Disease** | **Bisphosphonate** | **Dose(mg)** | **Duration** | **Group** |
| --- | --- | --- | --- | --- | --- | --- | --- |
| **Patient 1** | 37 | Female | Maxillary protrusion,  Mandibular retrusion | No | 0 | 0 | Control |
| **Patient 2** | 25 | Female | Maxillary retrusion,  Mandibular protrusion | No | 0 | 0 | Control |
| **Patient 3** | 21 | Male | Maxillary retrusion,  Mandibular protrusion | No | 0 | 0 | Control |
| **Patient 4** | 34 | Female | Maxillary retrusion,  Mandibular protrusion | No | 0 | 0 | Control |
| **Patient 5** | 27 | Female | Maxillary retrusion,  Mandibular protrusion | No | 0 | 0 | Control |
| **Patient 6** | 54 | Male | Lung cancer | Zoledronic acid | 4(/month) | 27 months | BRONJ |
| **Patient 7** | 65 | Female | Lung cancer | Zoledronic acid | 4(/1-3months) | 24 months | BRONJ |
| **Patient 8** | 66 | Female | Lung cancer | Zoledronic acid | 4(/month) | 36 months | BRONJ |
| **Patient 9** | 72 | Female | Multiple myeloma | Zoledronic acid | 4(/2months) | 48 months | BRONJ |
| **Patient 10** | 61 | Male | Lung cancer | Ibandronic acid | 4(/month) | 25 months | BRONJ |

**Table S2.** Primers of rabbit for Gene Expression Analysis

| Gene | Primer Sequences (5’-3’) |
| --- | --- |
| ACTIN- forward | CATGTACGTTGCTATCCAGGC |
| ACTIN- reverse | CTCCTTAATGTCACGCACGAT |
| TGF-β1- forward | TGTCACTGGAGTTGTGAGGC |
| TGF-β1- reverse | AGCAGTTCTTCTCTGTGGAGC |

**Table S3.** Primers of mouse for Gene Expression Analysis

| Gene | Primer Sequences (5’-3’) |
| --- | --- |
| ACTIN- forward | TGCTGTCCCTGTATGCCTCT |
| ACTIN- reverse | TTGATGTCACGCACGATTTC |
| DC STAMP -forward | CGAAGCTCCTTGAGAAACGA |
| DC STAMP -reverse | GGACTGGAAACCAGAAATGAA |
| cathepsin K- forward | CGAAAAGAGCCTAGCGAACA |
| cathepsin K- reverse | TGGGTA- GCAGCAGAAACTTG |
| Atp6v0d2- forward | AAGCCTTTGTTTGACGCTGT |
| Atp6v0d2- reverse | GCCAGCA CATTCATCTGTACC |
