# Supplementary material for: Adipose-Derived Stem Cells Promote Bone Coupling in Bisphosphonate-Related Osteonecrosis of the Jaw by TGF-β1
Source: Front Cell Dev Biol. 2021 May 12;9:639590. doi: 10.3389/fcell.2021.639590 (PMC8154543; doi:10.3389/fcell.2021.639590)
Supplement: Supplementary file 2 [file Data_Sheet_2.docx]

**Supplemental File 2**

**Supplementary Materials and Methods**

**Induction of BRONJ-like animal model.**

In brief, Rabbits from the zoledronate-treated groups received intravenous (IV) zoledronate (Zometa, 800ug/kg; Sigma, SML0223, USA) and dexamethasone (10 mg/kg; Shanghai Chemical Reagent Co. Ltd., China) once a week via the ear vein. Six weeks after the IV injection, bilateral maxillary premolars were extracted under deep anesthesia and received zoledronate injection for two more weeks. Rabbits from the healthy control group received saline instead of zoledronate. Two and eight weeks after tooth extraction, the maxilla was harvested. Bone formation was analyzed by cone beam computer tomography (CBCT) (J. Morita Corp., Kyoto, Japan) with 60 kV and 2 mA and histology. One side of the maxilla was collected to analyze TGF-β1 mRNA expression, while the other side was used for histological analysis.

**Immunochemistry.**

Anti-TGF-β1 antibody (R&D, MAB240, USA) was used for animal samples and anti-TGF-β1 antibody (Abcam, ab27969, USA) was used for human samples. Sample sections (4μm) were deparaffinized, rehydrated, and treated with trypsin (Abcam, ab970, USA) for antigen retrieval. After peroxidase elimination, samples were blocked with 5% (v/v) goat serum. Subsequently, samples were incubated with primary antibodies (1:200 dilution for anti-Runx2, 1:500 dilution for anti-TGF-β1) in goat serum at 4°C overnight, followed by goat anti-mouse secondary antibody (1:100) labeled with HRP for 20 min, stained with a 3,3′–diaminobenzidine kit (Vector Labs, UK) for 2.5 min, and counter-stained with hematoxylin. All stained sections were dehydrated and photographed in 3 random fields around the alveolar sockets in each sample and the mean quantified value was used for statistical analysis. The TGF-β1 positive areas were quantified by ImageJ software (National Institutes of Health). The Runx2 positive bone lining cells (BLCs) per bone marrow circumference were counted as osteoclasts.

**Collection of ADSCs-CM.**

To collect the CM, ADSCs were first cultured in 10% FBS-α-MEM to 70% confluency and then incubated in serum-free medium (F12/DMEM, Gibco) for 72 hours. The medium was collected and cleared for 5 min at 300× g, and filtered through a 0.22μm syringe filter. The collected ADSCs-CM at passage 3-5 was subdivided in vials and cryopreserved at -80℃ for subsequent use. ADSCs‐CM or F12/DMEM (Gibco) was used with HA for local implantation into extraction sites for in vivo studies. For osteoclastogenesis and the in vitro BMSCs migration assay, equal volumes of ADSCs‐CM and F12/DMEM (Gibco) were used. To verify the effects of TGF-β1 on ADSCs-CM-activated bone coupling in BRONJ-like animals, TGF-β1 neutralizing antibody (R&D, MAB240, USA) was added to ADSCs-CM with HA before implantation.
